# Forward Left Ventricular Ejection Fraction as a Predictor of Postoperative Left Ventricular Dysfunction in Patients with Degenerative Mitral Regurgitation

**DOI:** 10.3390/jcm10143013

**Published:** 2021-07-06

**Authors:** Juyoun Kim, Jae-Sik Nam, Youngdo Kim, Ji-Hyun Chin, In-Cheol Choi

**Affiliations:** Department of Anesthesiology and Pain Medicine, Asan Medical Center, University of Ulsan College of Medicine, Seoul 05505, Korea; juyoun1012@naver.com (J.K.); jaesik_nam@naver.com (J.-S.N.); midorimd@naver.com (Y.K.); icchoi@amc.seoul.kr (I.-C.C.)

**Keywords:** degenerative mitral regurgitation, forward left ventricular ejection fraction, mitral valve repair, postoperative left ventricular dysfunction

## Abstract

Background: Left ventricular dysfunction (LVD) can occur immediately after mitral valve repair (MVr) for degenerative mitral regurgitation (DMR) in some patients with normal preoperative left ventricular ejection fraction (LVEF). This study investigated whether forward LVEF, calculated as left ventricular outflow tract stroke volume divided by left ventricular end-diastolic volume, could predict LVD immediately after MVr in patients with DMR and normal LVEF. Methods: Echocardiographic and clinical data were retrospectively evaluated in 234 patients with DMR ≥ moderate and preoperative LVEF ≥ 60%. LVD and non-LVD were defined as LVEF < 50% and ≥50%, respectively, as measured by echocardiography after MVr and before discharge. Results: Of the 234 patients, 52 (22.2%) developed LVD at median three days (interquartile range: 3–4 days). Preoperative forward LVEF in the LVD and non-LVD groups were 24.0% (18.9–29.5%) and 33.2% (26.4–39.4%), respectively (*p* < 0.001). Receiver operating characteristic (ROC) analyses showed that forward LVEF was predictive of LVD, with an area under the ROC curve of 0.79 (95% confidence interval: 0.73–0.86), and an optimal cut-off was 31.8% (sensitivity: 88.5%, specificity: 58.2%, positive predictive value: 37.7%, and negative predictive value: 94.6%). Preoperative forward LVEF significantly correlated with preoperative mitral regurgitant volume (correlation coefficient [CC] = −0.86, *p* < 0.001) and regurgitant fraction (CC = −0.98, *p* < 0.001), but not with preoperative LVEF (CC = 0.112, *p* = 0.088). Conclusion: Preoperative forward LVEF could be useful in predicting postoperative LVD immediately after MVr in patients with DMR and normal LVEF, with an optimal cut-off of 31.8%.

## 1. Introduction

Degenerative mitral regurgitation (DMR) is the second most common valvular heart disease [1]. Mitral valve surgery is recommended before the onset of left ventricular (LV) dysfunction (LVD) [2], primarily because preoperative LVD has been associated with unfavorable outcomes [3,4,5], and early mitral valve surgery was found to be associated with a greater long-term survival benefit and a lower risk of heart failure than medical treatment [6].

LVD has been observed after mitral valve surgery for DMR, even in patients with normal preoperative LV function, with studies attempting to identify preoperative echocardiographic parameters that are associated with postoperative LVD [7,8,9,10,11,12]. Studies assessing factors that correlate with postoperative LVD occurring immediately after mitral valve surgery have identified that LV ejection fraction (LVEF), LV end-systolic diameter (LVESD), preoperative right ventricular systolic pressure and global longitudinal strain may be associated with immediately postoperative LVD [7,8,9,10,11,12,13]. A previous report demonstrated that only about one-third of patients with LVD immediately after surgery experienced recovery of LV function during long-term follow-up [7]. Therefore, identification of factors predictive of immediately postoperative LVD may be clinically significant, in that it may help to determine patients requiring early surgery to minimize LVD occurrence.

Forward LVEF, calculated as forward stroke volume (SV) divided by LV end-diastolic volume (LVEDV), may helpful in stratifying the long-term risk in patients with ≥ mild MR [14]. Few studies to date have evaluated the ability of forward LVEF to predict short-term risk, especially in terms of LVD after mitral valve surgery. The present study investigated whether forward LVEF could predict LVD immediately after mitral valve repair (MVr) in patients with DMR and normal preoperative LVEF.

## 2. Materials and Methods

### 2.1. Study Population

This retrospective observational study included patients who underwent MVr for DMR with grade ≥ moderate, at Asan Medical Center (Seoul, Korea) from January 2010 to December 2018. Patients were excluded if they had preoperative LVEF < 60% or coronary artery disease; if forward LVEF could not be calculated because of limited data; if they had undergone redo mitral valve surgery; or if immediately postoperative echocardiography showed remnant MR with grade ≥ moderate. The research protocol was approved by our Institutional Review Board (AMC IRB 2020–1918), which waived the requirement for written informed consent because of the retrospective nature of the study. Data were acquired from a retrospective review of electronic medical records.

### 2.2. Echocardiographic Data

All patients underwent transthoracic echocardiographic examination before and after MVr. Our institution followed the standards and techniques recommended by the American Society of Echocardiography for measuring MR severity [15,16]. Two-dimensional echocardiography and Doppler color flow imaging were performed in all patients using a Hewlett-Packard Sonos 2500, 5500, or 7500 imaging system (Hewlett-Packard, Andover, MA, USA) and a VIVID 7 or E9 ultrasound system (General Electric Healthcare, Little Chalfont, UK) with a 2.5 MHz probe. The left ventricular end-diastolic diameter and LV end-systolic diameter (LVESD) were measured from parasternal M-mode acquisitions, and the LV end-systolic volume (LVESV) and LVEDV were measured using the biplane Simpson method. LVEF was calculated from the measured LVESV and LVEDV. Measurements were averaged over three to five cardiac cycles for patients with atrial fibrillation.

Comprehensive echocardiographic evaluation of mitral regurgitation was performed using an integrated approach including 2-dimensional, Doppler, and color flow imaging. The proximal isovelocity surface area (PISA) was determined by measuring the proximal flow convergence by lowering the imaging depth and reducing the Nyquist limit at mid-systole. Various views were evaluated for optimal visualization of the PISA. Baseline shift was used to adjust the aliasing velocity to about 40 cm/s.

Forward LVEF was calculated using the following equation: forward LVEF = 100 × forward SV/LVEDV, where the forward SV was measured by pulsed wave Doppler in the LV outflow tract [14]. To determine intra-observer and inter-observer variability, a random sample of 25% of left ventricular outflow tract (LVOT) recordings was submitted twice to the first investigator and once to a second investigator. The inter-observer variability was calculated as the mean absolute difference between the two readings from the first and the second investigator divided by their mean. Similarly, the intra-observer variability was calculated as the mean absolute difference between the two readings from the first investigator divided by their mean.

In addition, we calculated the midwall fractional shortening (mFS) to assess LV contractility. The actual midwall fractional shortening (mFS) was determined using the two-shell method of Shimizu et al. [17]. Circumferential end-systolic stress (cESS), a measure of ventricular afterload, was calculated at the midwall according to the method of Gaasch et al. [18]. Thereafter, the predicted mFS was determined for any given cESS using the regression equation derived from a healthy population [19]. To minimize afterload dependence, stress-corrected mFS (sc-mFS) was calculated as the ratio of actual to predicted mFS [19].

Preoperative echocardiographic data were those determined closest to the day of surgery, and postoperative echocardiographic data were those determined before discharge without significant inotropic support or a mechanical assist device. The postoperative period was defined as after MVr and before hospital discharge.

### 2.3. Statistical Analysis

Continuous data are presented as mean ± standard deviation (SD) or as median (interquartile range [IQR]), and categorical data are presented as frequencies (percentages). The LVD and non-LVD groups were defined as patients with LVEF < 50% and ≥50%, respectively, as measured by echocardiography after MVr and before discharge. Continuous data in the LVD and non-LVD groups were compared by Student’s t-test or Mann– Whitney U test, and categorical data by Chi-square test or Fisher’s exact test, as appropriate. Within group echocardiographic parameters measured preoperatively and immediately postoperatively were compared by paired t-test or Wilcoxon signed rank test, as appropriate.

Receiver operating characteristic (ROC) curve analyses were performed to determine the performance of individual parameters for predicting LVD. The optimal cut-off on ROC curves was defined as the value based on Youden’s Index, which was calculated as maximum (sensitivity + specificity − 1). Correlations of forward LVEF, mitral regurgitant volume (RVol), mitral regurgitant fraction (RF), and LVEF were calculated using Pearson or Spearman correlation analyses. *p* < 0.05 was considered statistically significant. We conducted analyses using the SigmaPlot 13.0 (Systat Software Inc., San Jose, CA, USA).

## 3. Results

During the study period, 706 patients underwent MVr for DMR. Of these, there were 472 patients who did not satisfy the inclusion criteria as follows: 30 with preoperative LVEF < 60%, 9 with coronary artery disease, 427 who did not have available data for calculating forward LVEF, 1 who underwent redo mitral valve surgery, 4 with MR ≥ moderate at immediately postoperative echocardiography, and 1 with inotropic support during immediately postoperative echocardiographic examination. Thus, the remaining 234 patients were evaluated.

Patients underwent immediate postoperative echocardiography three (3–4) days after MVr. Of 234 patients, 52 (22.2%) experienced immediately postoperative LVD. The preoperative demographic characteristics, comorbidities, and medications are presented in Table 1. Chronic kidney disease and atrial fibrillation were more prevalent in the LVD than in the non-LVD group. By contrast, age, the proportion of male, Euroscore Ⅱ, preoperative medications, and cardiopulmonary bypass and aortic cross clamping times were similar in the two groups.

A comparison of preoperative echocardiographic parameters in the two groups showed that preoperative LVEF, forward SV, and forward LVEF were lower in the LVD than in the non-LVD group, whereas preoperative LVESD, LVEDV, LV mass index, RVol and RF were higher in the LVD than in the non-LVD group (Table 2).

The inter-observer and intra-observer variabilities for LVOT measurement were 5.0 ± 2.9% and 4.3 ± 2.7%, respectively.

Differences in echocardiographic parameters measured preoperatively and immediately postoperatively were also assessed in the two groups. LVEDV, LVEF, mFS, and sc-mFS were lower postoperatively than preoperatively in both groups, whereas cESS was lower postoperatively only in the non-LVD group (Table 3). Between-group comparisons of postoperative parameters showed that postoperative LVEDV was lower, whereas postoperative sc-mFS was higher, in the non-LVD than in the LVD group (Table 3).

**Figure 1:**
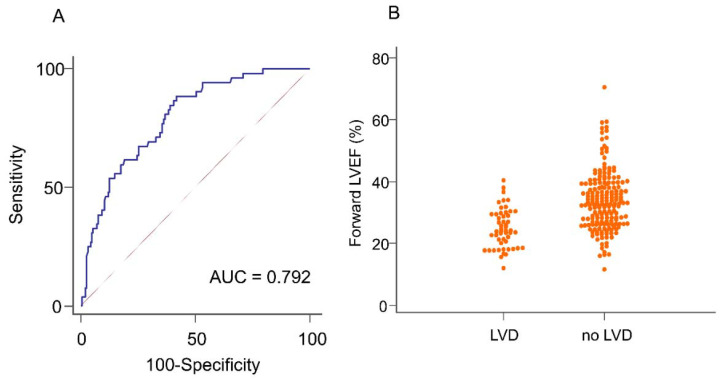
(**A**) Receiver operating characteristic curves demonstrating the performance of preoperative forward left ventricular ejection fraction (LVEF) to predict immediately postoperative left ventricular dysfunction (LVD), and (**B**) individual values of preoperative forward LVEF in the LVD and non-LVD group.

ROC analyses showed that forward LVEF was predictive of LVD, with an area under the ROC curve of 0.79 (95% confidence interval: 0.73–0.86). The optimal cut-off of 31.8% had a sensitivity of 88.5%, a specificity of 58.2%, a positive predictive value (PPV) of 37.7%, and a negative predictive value (NPV) of 94.6% (Figure 1, Table 4). The area under the ROC curve of LVESD was 0.75 (95% CI: 0.67–0.83), with an optimal cut-off of 38 mm (sensitivity of 65.4%, specificity of 74.2%, PPV of 42.2%, and NPV of 88.2%) (Table 4).

**Figure 2:**
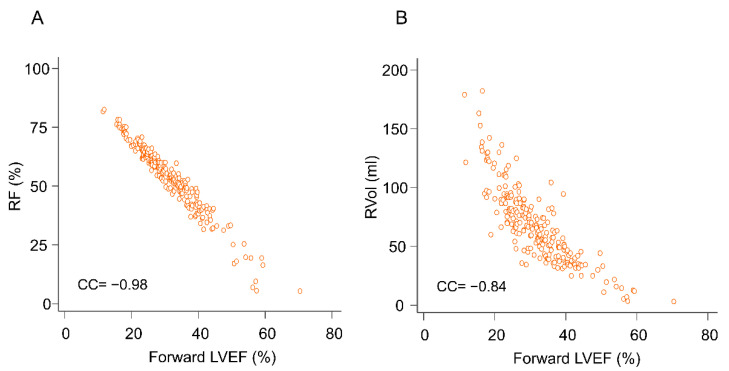
Correlations (**A**) between forward left ventricular ejection fraction (LVEF) and mitral regurgitant fraction (RF), and (**B**) between forward LVEF and mitral regurgitant volume (RVol).

In addition, preoperative forward LVEF correlated significantly with preoperative RVol (correlation coefficient [CC] = −0.86, *p* < 0.001) and RF (CC = −0.98, *p* < 0.001) (Figure 2), but not with preoperative LVEF (CC = 0.112, *p* = 0.088) and peak systolic velocity of mitral annulus (CC = 0.04, *p* = 0.529).

There were 62 patients (16 in the LVD group and 46 in the non-LVD group) who had available LVEF data at six months after MVr. LVEFs at six months were 55.5% (51.3–59.8%) and 60.5% (57.8–65.0%) in the LVD group and the non-LVD group, respectively (*p* < 0.001).

Between 6 and 18 months after MVr, LVEF data were available for 212 of the 234 included patients. Of these 212 patients, three (1.4%), all in the LVD group, showed LVEF < 50% at 6–18 months.

## 4. Discussion

The present study showed that preoperative forward LVEF could predict LVD immediately after MVr, with an optimal cut-off of 31.8% in patients with DMR and preoperative LVEF ≥ 60%. Compared with preoperative LVESD, preoperative forward LVEF showed a similar AUC on ROC analysis, but a higher NPV for predicting postoperative LVD.

Although many previous studies have evaluated postoperative LVD after MVr, few studies have investigated postoperative LVD occurring immediately after mitral valve surgery [7,11]. High right ventricular systolic pressure and LVESD were found to be independently associated with an increased likelihood of postoperative LVEF < 40%, with the latter being associated with increased mortality in patients with DMR and preoperative LVEF ≥ 60% who underwent MVr [7]. In addition, global longitudinal strain was found to be associated with postoperative LVEF reduction > 10% immediately after MVr for DMR [11].

Assessment of LV systolic function in asymptomatic patients with DMR is regarded as important, because mitral valve surgery is recommended before onset of LV systolic dysfunction [2]. Preoperative LVEF is regarded as indicative of LV systolic dysfunction, although LVEF could not be reflective of LV function because of a mechanism that compensated for volume overload of the left ventricle in patients with MR. Previous studies reported LV dysfunction present in patients with DMR and normal preoperative LVEF [20,21,22,23,24]. Marked myofibrillar degeneration and myocardial fibrosis, indicating LV contractile dysfunction, have been observed in patients with preoperative LVEF ≥ 60% before surgery. LV fibrosis was found to be more prevalent in patients with than without MV prolapse at all levels of MR fraction [20]. Moreover, diffuse interstitial fibrosis was a prevalent finding, occurring prior to any conventional class Ⅰ indications for mitral valve surgery. This finding suggested that MR did not have a physiological compensatory phase and that volume overload may lead to pathological changes in LV structure and function at an earlier stage [21]. LV dysfunction may be masked by normal, even supra-normal, LVEF, with this latent LV dysfunction revealed in the absence of overload [14]. The actual stage of preoperative LV function may be reflected by immediately postoperative LVEF [12,14], as surgical correction results in the disappearance of the confounding effect of mitral regurgitant volume, although the effects of changes in preload and afterload cannot be completely excluded.

Forward LVEF was found to be independently associated with long-term outcome, composite of mitral valve surgery and death, in patients with ≥mild MR, and may be superior to LVEF and LVESD in predicting outcomes [14]. That study also demonstrated that forward LVEF could predict postoperative LVD, with a cut-off value of 40% [14]. That study, however, did not specify the preoperative LVEF of studied patients and the timing of LVD occurrence. Another study reported that preoperative forward LVEF < 40% could predict LVD three months after surgery in a small number of patients undergoing mitral valve surgery [25]. In our study, forward LVEF could predict LVD immediately after MVr, with an optimal cut-off of 31.8% in patients with DMR and preoperative LVEF ≥ 60%. When compared with conventional parameters, the AUCs for preoperative forward LVEF and preoperative LVESD were comparable, but NPV was higher for preoperative forward LVEF than for preoperative LVESD. The NPV of forward LVEF was also higher than that of LVESD, RVol, and RF. The NPV result indicated that 94.6% of patients with preoperative forward LVEF > 31.8% would truly not have postoperative LVD, suggesting that forward LVEF may be a useful parameter in the follow-up of patients with DMR and normal LVEF. Moreover, forward LVEF may be the most helpful parameter in the follow-up to detect latent LV dysfunction before mitral valve surgery in patients with DMR. Because NPV increases as disease prevalence decreases [26], caution should be exercised when interpreting the results. However, as the prevalence of LVD in our study (22.2%) was not lower compared with that in a previous study (18%) [7], the clinical significance of our result may not be attenuated.

LVEF decreased more in the LVD than in the non-LVD group. LVEF is a parameter that could be affected by changes in LV preload and afterload, as well as by intrinsic LV contractility. The decrease in LVEDV (preload) from before to immediately after surgery was greater in the LVD than in the non-LVD group, whereas cESS (afterload) increased only in the LVD group. These findings may contribute, at least in part, to the greater decrease in postoperative LVEF in the LVD group. By contrast, postoperative sc-mFS, an indicator of afterload-adjusted LV contractility, was higher, despite postoperative LVEDV being lower, in the non-LVD than in the LVD group. These findings suggest that LV intrinsic contractility may be more impaired in the LVD than the non-LVD group, and that this latent LV dysfunction may be revealed postoperatively. Although postoperative LVD may be due to myocardial stunning, the latter typically resolves 48–72 h after ischemia. Because our patients underwent immediate postoperative echocardiographic examination which was performed at median three days (3–4 days) after surgery, myocardial stunning was an unlikely cause of postoperative LVD in the present study. This was supported by our results showing that cardiopulmonary bypass and aortic cross clamping time were not different between the two groups.

Left atrial enlargement has been reported to be a predictor of common cardiovascular outcomes such as atrial fibrillation [27]. In our data, the left atrium was enlarged in both groups, with more enlargement in the LVD group and, therefore, a higher incidence of atrial fibrillation in the LVD group than in the non-LVD group. Moreover, a larger left atrial dimeter indicates a higher left ventricular end-diastolic pressure in the LVD group than that in the non-LVD group. It has been reported that myocardial fibrosis developed in patients with MR, especially with DMR [20,21,28], and the severity of myocardial fibrosis was associated with the degree of LV filling pressure [29]. Based on these previous observations, we speculate that our result on a higher incidence of atrial fibrillation in the LVD group suggests the possibility that more myocardial fibrosis may exist in the LVD group than in the non-LVD group. Further studies need to confirm this association.

Our results also showed that preoperative forward LVEF was significantly inversely correlated with RVol and RF, suggesting that forward LVEF may reflect the degree of MR, not LV systolic function. Increased RVol results in greater damage to the left ventricle, and implies a longer exposure of the left ventricle to volume overload by MR, as MR can foster a greater MR. A lower forward LVEF may reflect a greater regurgitant amount of MR, followed by more impaired LV function, which could be revealed in the absence of compensation mechanisms for overloaded volume immediately after MVr, in MR patients with normal or even supra-normal preoperative LVEF.

In addition, we found that LVEFs at six months in the non-LVD group were still higher than in the LVD group. Compared with the LVEF immediately after surgery, the LVEF measured at six months improved in both groups, with more improvement in the LVD group than in the non-LVD group. This may, at least in part, be attributed to differences in changes in the loading conditions of the left ventricle and LV reverse remodeling after MVr. Further study of long-term trajectories of LV function using global longitudinal strain is needed in both groups to confirm this issue. We also found that three patients who experienced LVD immediately after surgery also experienced LVEF < 50% 6−18 months after surgery. These findings suggest a need for additional criteria to distinguish patients who will and will not show resolution of LVD immediately after surgery. Because the number of patients was limited, however, we could not determine the significance of sustained LVEF < 50% 6–18 months after surgery.

This study had several limitations. First, some technical limitations of measuring SV using Doppler echocardiography could not be completely excluded, although experienced sonographers performed echocardiographic examination in our high-volume institution. LVOT diameter error is frequently referred to as the most common potential source of error for SV measurement by Doppler echocardiography, because it amplifies the measurement error due to the square of the LVOT diameter in continuity equation [30]. In addition, Doppler echocardiography has the limitation of underestimation of the flow velocity when the ultrasound beam is not parallel to the flow at LVOT. Second, this study had a retrospective observational design, with its associated inherent limitations. Third, because of limited data, we did not evaluate whether LVD occurring immediately after MVr was associated with a decreased LVEF 6–18 months after surgery, or with the long-term outcomes. Additional studies in larger patient cohorts are warranted.

In conclusion, the present study showed that preoperative forward LVEF with cut-off value of 31.8% could predict immediately postoperative LVD after MVr in patients with DMR and normal preoperative LVEF. Our results suggest that forward LVEF may be a useful parameter to distinguish DMR patients with and without preoperative latent LV dysfunction.

## Figures and Tables

**Table 1:** Baseline characteristics.

	Total	LVD	Non-LVD	*p*
**Demographics**				
Age (years)	51.8 ± 11.8	52.2 ± 10.8	51.7 ± 12.1	0.781
Male	165 (70.5)	37 (71.2)	127 (69.8)	1.0
BMI (kg/m^2^)	24.7 (22.5–26.5)	24.3 (21.6–25.9)	24.7 (22.7–26.7)	0.113
**Comorbidities**				
DM	41 (17.5)	12 (23.1)	29 (15.9)	0.3
HTN	93 (39.7)	20 (38.5)	73 (40.1)	0.873
CVA	5 (2.1)	0 (0)	5 (2.7)	0.589
PVD	2 (0.9)	0 (0)	2 (1.1)	1.0
COPD	7 (3.0)	0 (0)	7 (3.8)	0.353
CKD	6 (2.6)	3 (5.8)	3 (1.6)	0.005
A-fib	26 (11.1)	11 (21.2)	15 (8.2)	0.021
Euroscore Ⅱ	0.7 (0.5–1.0)	0.7 (0.6–1.0)	0.7(0.5–1.0)	0.445
**Preoperative Medication**				
ACEI/ARB	112 (47.9)	26(50.0)	86 (47.3)	0.755
β-blocker	68 (29.1)	16 (30.8)	52 (28.6)	0.733
CCB	52 (22.2)	13 (25.0)	39 (21.4)	0.575
Digoxin	15 (6.4)	3 (5.8)	12 (6.6)	1.0
Diuretics	107 (45.7)	25 (48.1)	82 (45.1)	0.753
**Intraoperative Data**				
Op time (mins)	275.0 (237.0–323.3)	256.5 (233.3–305.0)	280.0 (237.0–325.0)	0.187
CPB time (mins)	140.0 (115.8–170.0)	144.5 (113.0–167.5)	138.0 (115.8–170.5)	0.679
ACC time (mins)	77.5 (64.0–99.0)	84.0 (71.3–107.0)	77.0 (63.0–96.0)	0.093
Colloid (L)	0.5 (0.3–0.5)	0.5 (0.4–0.5)	0.5 (0.3–0.5)	0.095
Crystalloid (L)	1.5 (1.2–1.8)	1.5 (1.0–1.6)	1.5 (1.2–1.8)	0.073

Data are presented as mean ± SD, median (interquartile range), or number (percentage). The LVD and non-LVD groups were defined as patients with left ventricular ejection fraction < 50% and ≥50%, respectively, as measured by echocardiography after mitral valve repair and before discharge. BMI = body mass index; DM = diabetes mellitus; HTN = hypertension; CVA = cerebrovascular accident; PVD = peripheral vascular disease; COPD = chronic obstructive pulmonary disease; CKD = chronic kidney disease; A-fib = atrial fibrillation; ACEI = angiotensin converting enzyme inhibitor; ARB = angiotensin receptor blocker; CCB = calcium channel blocker; CPB = cardiopulmonary bypass; ACC = aorta cross clamping.

**Table 2:** Preoperative echocardiographic findings.

	Total	LVD	Non-LVD	*p*
LVEF	68.0 (65.0–71.0)	66.5 (64.0–69.0)	68.0 (65.0–72.0)	0.020
LVESD	36.7 ± 5.2	40.3 ± 4.9	35.6 ± 4.8	<0.001
LVEDD	59.6 ± 5.5	63.8 ± 4.5	58.4 ± 5.1	<0.001
LVESV	56.0 (43.0–68.0)	70.5 (58.0–80.0)	51.5 (41.0–62.3)	<0.001
LVEDV	172.0 (141.8–209.3)	210.5 (184.3–238.5)	163.0 (137.0–197.0)	<0.001
RWT	0.32 (0.29–0.35)	0.30 (0.27–0.32)	0.33 (0.29–0.36)	<0.001
LVMI	129.3 (112.3–151.6)	149.6 (133.3–168.5)	123.5 (108.5–141.9)	<0.001
LAD	47.0 (43.0–53.0)	49.5 (46.3–55.8)	46.0 (43.0–52.0)	<0.001
PG_TR_	29.0 (23.0–36.0)	29.0 (27.0–38.0)	27.0 (23.0–36.0)	0.104
E/A	2.0 (1.5–2.5)	2.2 (1.7–2.5)	1.9 (1.4–2.6)	0.303
S′	7.9 (7.0–8.7)	7.9 (7.1–8.9)	7.9 (7.0–8.7)	0.568
E′	8.0 (6.4–9.9)	8.3 (6.3–9.6)	8.0 (6.5–10.0)	0.897
A′	7.5 (6.2–8.9)	7.1 ± 1.7	7.6 ± 1.9	0.098
E/E′	14.0 (11.0–19.0)	15.5 (11.3–19.8)	14.0 (11.0–19.0)	0.234
cESS	135.8 (113.2–161.3)	154.0 (129.8–170.5)	131.9 (112.2–157.9)	0.006
mFS	20.3 (18.6–21.9)	20.2 (18.7–21.9)	20.3 (18.5–21.9)	0.843
sc-mFS	118.9 (109.5–130.4)	123.7 (111.9–131.3)	118.2 (108.3–130.4)	0.114
Forward SV	53.1 (45.0–60.6)	50.1 (43.3–59.2)	54.0 (46.0–61.8)	0.074
Forward LVEF	31.4 (24.9–37.4)	24.0 (18.9–29.5)	33.2 (26.8–39.4)	<0.001
EROA	0.8 (0.6–1.1)	1.0 (0.8–1.3)	0.8 (0.6–1.1)	<0.001
RVol	64.5 (44.0–86.4)	85.7 (69.6–110.8)	57.5 (39.3–78.0)	<0.001
RF	54.0 (45.4–62.7)	62.7 (54.4–70.1)	51.8 (41.5–60.0)	<0.001

Data are presented as mean ± SD or median (interquartile range). The LVD and non-LVD groups were defined as patients with LVEF < 50% and ≥50%, respectively, as measured by echocardiography after mitral valve repair and before discharge. LVEF = left ventricular ejection fraction; LVESD = left ventricular end-systolic diameter; LVEDD = left ventricular end-diastolic diameter; LVESV = left ventricular end-systolic volume; LVEDV = left ventricular end-diastolic volume; RWT = relative wall thickness; LVMI = left ventricular mass index; LAD = left atrial diameter; PG_TR_= pressure gradient calculated from peak tricuspid regurgitation; E/A = ratio of peak early and late diastolic velocity of mitral inflow; S′ = peak systolic velocity of mitral annulus; E′ = peak early diastolic velocity of mitral annulus; A′ = peak late diastolic velocity of mitral annulus; E/E′ = ratio of peak early diastolic velocity of mitral inflow to mitral annulus early diastolic velocity; cESS = circumferential end-systolic stress; mFS = midwall fractional shortening; sc-mFS = stress-corrected midwall fractional shortening; SV = stroke volume; EROA = effective regurgitant orifice area; RVol = regurgitant volume of mitral regurgitation; RF = regurgitant fraction of mitral regurgitation.

**Table 3:** Differences in echocardiographic findings between preoperative and immediately after mitral valve repair.

	LVD			Non-LVD				
	Pre	Post	*p* *	Pre	Post	*p* *	*p* ^†^	*p* ^‡^
LVEDV (preload)	210.5 (184.3–238.5)	141.5 (119.3–187.3)	<0.001	163.0 (137.0–197.0)	114.0 (95.8–137.0)	<0.001	<0.001	<0.001
cESS (afterload)	154.0 (129.8–170.5)	151.3 (133.4–186.7)	0.122	131.9 (112.2–157.9)	120.8 (101.9–138.5)	<0.001	0.006	<0.001
LVEF	66.5 (64.0–69.0)	45.0 (41.0–48.8)	<0.001	68.0 (65.0–72.0)	58.0 (55.0–62.0)	<0.001	0.020	<0.001
S′	7.9 (7.1–8.9)	5.5 (4.8–6.3)	<0.001	7.9 (7.0–8.7)	6.8 (5.9–7.9)	<0.001	0.568	<0.001
mFS	20.2 (18.7–21.9)	13.4 (12.1–15.3)	<0.001	20.3 (18.5–21.9)	16.0 (14.4–17.5)	<0.001	0.843	<0.001
sc-mFS	123.7 (111.9–131.3)	82.5 (72.3–92.0)	<0.001	118.2 (108.3–130.4)	91.9 (84.9–100.2)	<0.001	0.114	<0.001

Data are presented as median (interquartile range). The LVD and non-LVD groups were defined as patients with LVEF < 50% and ≥ 50%, respectively, as measured by echocardiography after mitral valve repair and before discharge. Pre= preoperative; Post= immediately postoperative; LVEDV = left ventricular end-diastolic volume; cESS = circumferential end-systolic stress; LVEF = left ventricular ejection fraction; S′ = peak systolic velocity of mitral annulus; mFS = midwall fractional shortening; sc-mFS = stress-corrected midwall fractional shortening. * *p* < 0.05 in comparison of parameters between preoperative and postoperative period in each group. ^†^ *p* < 0.05 in comparison of preoperative parameters between LVD and non-LVD groups. ^‡^ *p* < 0.05 in comparison of postoperative parameters between LVD and non-LVD groups.

**Table 4:** Predictive ability of echocardiographic parameters for LVD immediately after MVr.

	AUC (95% CI)	Cut-off	Sen (%)	Spe (%)	PPV (%)	NPV (%)
LVESD (mm)	0.75(0.67–0.83)	38.0	65.4	74.2	42.0	88.2
Forward LVEF (%)	0.79 (0.73–0.86)	31.8	88.5	58.2	37.7	94.6
RVol (mL)	0.78 (0.71–0.84)	73.5	73.1	70.3	41.3	90.1
RF (%)	0.77(0.70–0.84)	60.6	65.4	78.6	46.6	88.8
LVEDV (mL)	0.77 (0.70–0.84)	196.0	71.2	74.2	43.0	89.8
LVESV (mL)	0.78 (0.72–0.85)	55.0	86.5	58.8	36.0	93.6

LVD = left ventricular dysfunction; MVr = mitral valve repair; AUC = area under curve; CI = confidence interval; Sen = sensitivity; Spe = specificity; PPV = positive predictive value; NPV = negative predictive value; LVESD = left ventricular end-systolic diameter; LVEF = left ventricular ejection fraction; RVol = regurgitant volume of mitral regurgitation; RF = regurgitant fraction of mitral regurgitation; LVEDV = left ventricular end-diastolic volume; LVESV = left ventricular end-systolic volume.

## Data Availability

The data presented in this study are available on request from the corresponding author with a reasonable reason.
